# Causal inference with observational data: the need for triangulation of evidence

**DOI:** 10.1017/S0033291720005127

**Published:** 2021-03

**Authors:** Gemma Hammerton, Marcus R. Munafò

**Affiliations:** 1 Population Health Sciences, Bristol Medical School, University of Bristol, Bristol, UK; 2 MRC Integrative Epidemiology Unit at the University of Bristol, Bristol, UK; 3 School of Psychological Science, University of Bristol, Bristol, UK

**Keywords:** causal inference, epidemiology, mental health, observational data, triangulation

## Abstract

The goal of much observational research is to identify risk factors that have a causal effect on health and social outcomes. However, observational data are subject to biases from confounding, selection and measurement, which can result in an underestimate or overestimate of the effect of interest. Various advanced statistical approaches exist that offer certain advantages in terms of addressing these potential biases. However, although these statistical approaches have different underlying statistical assumptions, in practice they cannot always completely remove key sources of bias; therefore, using design-based approaches to improve causal inference is also important. Here it is the design of the study that addresses the problem of potential bias – either by ensuring it is not present (under certain assumptions) or by comparing results across methods with different sources and direction of potential bias. The distinction between statistical and design-based approaches is not an absolute one, but it provides a framework for *triangulation* – the thoughtful application of *multiple* approaches (e.g. statistical and design based), each with their own strengths and weaknesses, and in particular sources and directions of bias. It is unlikely that any single method can provide a definite answer to a causal question, but the triangulation of evidence provided by different approaches can provide a stronger basis for causal inference. Triangulation can be considered part of wider efforts to improve the transparency and robustness of scientific research, and the wider scientific infrastructure and system of incentives.

## What is a causal effect?

The goal of much observational research is to establish causal effects and quantify their magnitude in the context of risk factors and their impact on health and social outcomes. To establish whether a specific exposure has a causal effect on an outcome of interest we need to know what would happen if a person were exposed, and what would happen if they were not exposed. If these outcomes differ, then we can conclude that the exposure is causally related to the outcome. However, individual causal effects cannot be identified with confidence in observational data because we can only observe the outcome that occurred for a certain individual under one possible value of the exposure (Hernan, 2004). In a statistical model using observational data, we can only compare the risk of the outcome in those exposed, to the risk of the outcome in those unexposed (two subsets of the population determined by an individuals’ actual exposure value); however, inferring causation implies a comparison of the risk of the outcome if all individuals were exposed and if all were unexposed (the same population under two different exposure values) (Hernán & Robins, 2020). Inferring population causal effects from observed associations between variables can therefore be viewed as a missing data problem, where several untestable assumptions need to be made regarding bias due to confounding, selection and measurement (Edwards, Cole, & Westreich, 2015).

The findings of observational research can therefore be inconsistent, or consistent but unlikely to reflect true cause and effect relationships. For example, observational studies have shown that those who drink no alcohol show worse outcomes on a range of measures than those who drink a small amount (Corrao, Rubbiati, Bagnardi, Zambon, & Poikolainen, 2000; Howard, Arnsten, & Gourevitch, 2004; Koppes, Dekker, Hendriks, Bouter, & Heine, 2005; Reynolds et al., 2003; Ruitenberg et al., 2002). This pattern of findings could be due to confounding (e.g. by socio-economic status), selection bias (e.g. healthier or more resilient drinkers may be more likely to take part in research), reverse causality (e.g. some of those who abstain from alcohol do so because of pre-existing ill-health which leads them to stop drinking) (Chikritzhs, Naimi, & Stockwell, 2017; Liang & Chikritzhs, 2013; Naimi et al., 2017), or a combination of all of these. However, the difficulty in establishing generalizable causal claims is not simply restricted to observational studies. No single study or method, no matter the degree of excellence, can provide a definite answer to a causal question.

Approaches to causal inference may be broadly divided into two kinds – those that use statistical adjustment to control confounding and arrive at a causal estimate, and those that use design-based methods to do so. The former approaches rely on the assumption that there is no remaining unmeasured confounding and no measurement error after the application of statistical methods, while the latter does not. Effective statistical adjustment for confounding requires knowing what to measure – and measuring it accurately – whereas many design-based approaches [for example, randomized controlled trials (RCTs)] do not have that requirement. Approaches that rely on statistical adjustment are likely to have similar (or at least related) sources of bias, whereas those that rely on design-based methods are more likely to have different sources of bias. Although the distinction between statistical and design-based approaches is not absolute (all approaches require the application of statistical methods, for example), it nevertheless provides a framework for *triangulation.* That is, ‘*The practice of strengthening causal inferences by integrating results from several different approaches, where each approach has different* (*and assumed to be largely unrelated*) *key sources of potential bias*’ (Munafo & Davey Smith, 2018). No single approach can provide a definitive answer to a causal question, but the thoughtful application of multiple approaches (e.g. statistical and design based), each with their own strengths and weaknesses, and in particular sources and directions of bias, can provide a stronger basis for causal inference.

Although the concept of triangulation is not new, the specific, explicit application of this framework in the mental health literature is relatively limited and recent. Here we describe threats to causal inference, focusing on different sources of potential bias, and review methods that use statistical adjustment and design to control confounding and support the causal inference. We conclude with a review of how these different approaches, within and between statistical and design-based methods, can be integrated within a triangulation framework. We illustrate this with examples of studies that explicitly use a triangulation framework, drawn from the relevant mental health literature.

## Statistical approaches to causal inference

Three types of bias can arise in observational data: (i) *confounding bias* (which includes reverse causality), (ii) *selection bias* (inappropriate selection of participants through stratifying, adjusting or selecting) and (iii) *measurement bias* (poor measurement of variables in analysis). A glossary of italic terms is shown in Box 1.

Box 1. Glossary of terms

**Table:** 

*Backdoor pathway*. A non-causal path from the exposure to the outcome in a causal diagram that remains after removing all arrows pointing from the exposure to other variables *Causal diagram*. A graphical description that requires us to set down our assumptions about causal relationships between variables *Collider*. A common effect of two variables *Collider bias*. Conditioning (i.e. stratifying, adjusting or selecting) on a common effect of two variables which induces a spurious association between them within strata of the variable that was conditioned on (the collider) *Confounding bias*. Failure to condition on a third variable that influences both the exposure and the outcome, causing a spurious association between them *Counterfactual mediation*. The counterfactual approach to mediation is based on conceptualizing ‘potential outcomes’ for each individual [*Y*(*x*)] that would have been observed if particular conditions were met (i.e. had the exposure *X* been set to the value *x* through some intervention) – regardless of the conditions that were in fact met for each individual *Exclusion restriction criterion*. In MR, the assumption that the genetic variants only affect the outcome through their effect on the exposure *Latent variable*. A source of variance not directly measured but estimated from the covariation between a set of strongly related observed variables *Marginal structural models*. A class of statistical models used for causal inference with observational data that use inverse probability weighting to control for the effects of time-varying confounders that are also a consequence of a time-varying exposure *Measurement bias*. Errors in assessment of the variables in the analysis due to imprecise data collection methods *Missing data mechanism*. The process by which data are missing; MCAR means that the probability of variable *Z* being missing is not related to observed variables or true value of *Z* (i.e. cases with missing values can be regarded as a random sample); MAR means that the probability of *Z* being missing is not related to unobserved values of *Z* but may be related to observed *Z* and other observed variables; MNAR means that the probability of *Z* being missing still depends on unobserved values of *Z* even after allowing for dependence on observed values of *Z* and other observed variables *Overcontrol bias*. Conditioning on a variable on the causal pathway between the exposure and the outcome *Pleiotropy*. Genetic variants influence multiple traits; horizontal (or biological) pleiotropy occurs when a genetic variant directly and independently influences two or more traits, and is a threat to Mendelian randomization (MR), whereas vertical (or mediated) pleiotropy occurs when an effect on a downstream trait is mediated by an influence on an upstream trait, and is not a threat to MR *Population stratification*. Where systematic differences in both allele frequencies and traits of interest can give rise to spurious genetic associations *Propensity scores*. A score that is used to control for time-invariant confounding, calculated by estimating the probability that an individual is exposed, given the values of their observed baseline confounders *Regression discontinuity design*. In a situation where an intervention is provided to those who fall above (or below) a certain threshold on a specific measure, the outcome can be compared across individuals that fall just above and just below the threshold *Selection bias*. When the process used to select subjects into the study or analysis results in the association between the exposure and outcome in those selected differing from the association in the whole population *Triangulation*. The practice of strengthening causal inferences by integrating results from several different approaches, where each approach has different (and assumed to be largely unrelated) key sources of potential bias

These biases can all result from opening, or failing to close, a *backdoor pathway* between the exposure and outcome. Confounding bias is addressed by identifying and adjusting for variables that can block a backdoor pathway between the exposure and outcome, or alternatively, identifying a population in which the confounder does not operate. Selection bias is addressed by not conditioning on *colliders* (or a consequence of a collider), and therefore opening a backdoor pathway, or removing potential bias when conditioning cannot be prevented. Measurement bias is addressed by careful assessment of variables in analysis and, where possible, collecting repeated measures or using multiple sources of data. In Box 2 we outline each of these biases in more detail using *causal diagrams* – accessible introductions to causal diagrams are available elsewhere (Elwert & Winship, 2014; Greenland, Pearl, & Robins, 1999; Rohrer, 2018) – together with examples from the mental health literature.

Box 2. Threats to causal inference.

**Table:** 

**Figure 1.**
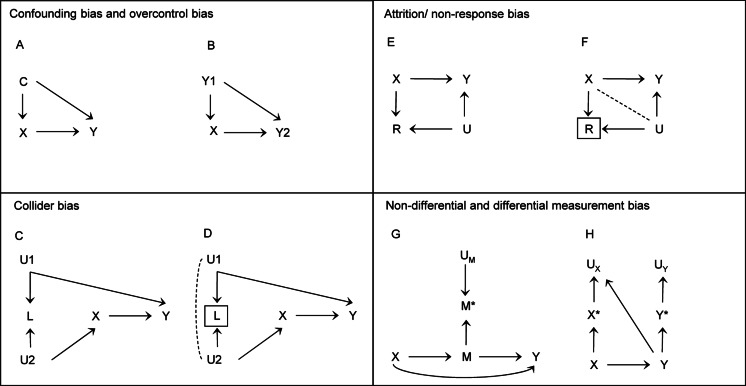
Causal diagrams representing confounding, selection bias and measurement bias *Note*: in the causal diagrams above, we assume that: (i) all observed and unobserved common causes in the process under investigation are displayed, (ii) there is no chance variation (i.e. we are working with the entire population), and (iii) the absence of an arrow represents no causal effect between variables. Additionally, to demonstrate selection bias, we also show diagrams with non-causal paths, where associations have been induced by conditioning on a common effect (or collider). Explanations of how biases due to confounding, selection and measurement can be described using potential outcomes are available elsewhere (Edwards et al., 2015; Hernan, 2004)

*Confounding and reverse causality.* A confounder is a third variable (*C*) that influences both the exposure (*X*) and the outcome (*Y*), causing a spurious association between them. Traditionally, a confounder was defined on the basis of three criteria, namely that it should be: (i) associated with *X*; (ii) associated with *Y*, conditional on *X* and (iii) not on the causal pathway between *X* and *Y*. For example, Fig. 1*A* shows the association between smoking (*X*) and educational attainment (*Y*), which is partly confounded by behavioural problems (*C*). Reverse causality is a specific case of confounding where pre-existing symptoms of the outcome can cause the exposure and result in the observed association between the exposure and outcome. Reverse causality is often addressed by adjusting for a baseline measure of the outcome (*Y*1) when examining the association between the exposure (*X*) and the outcome at follow-up (*Y*2). However, because *X* and *Y*1 are assessed simultaneously, it is possible that *Y*1 is on the causal pathway between *X* and *Y*2 (Fig. 1*B*) resulting in *overcontrol bias*. A second example of inappropriate adjustment for confounding follows directly from the traditional definition of a confounder. Figure 1*C* shows an example of a third variable (*L*) which is associated with the exposure (*X*) due to an unmeasured confounder (*U*2), and associated with outcome (*Y*) due to an unmeasured confounder (*U*1), and not on the causal pathway between *X* and *Y*. According to the traditional definition, *L* should be adjusted for in the analyses. However, as shown in Fig. 1*D*, conditioning on *L* (represented by a square drawn around *L*) induces an association between *U*1 and *U*2 (represented by a dashed line) which introduces unmeasured confounding for the association between *X* and *Y*. This is an example of collider bias, which is discussed in more detail below. A more recent definition of a confounder that prevents this potential bias occurring is a variable that can be used to block a backdoor path between the exposure and outcome (Hernan & Robins, 2020). *Selection bias.* Selection bias is an overarching term for many different biases including differential loss to follow-up, non-response bias, volunteer bias, healthy worker bias, and inappropriate selection of controls in case−control studies (Hernan, 2004). It is present when the process used to select subjects into the study or analysis results in the association between the exposure and outcome in those selected subjects differing from the association in the whole population (Hernan, Hernandez-Diaz, & Robins, 2004). This bias is (usually) a consequence of conditioning (i.e. stratifying, adjusting or selecting) on a common effect of an exposure and an outcome (or a common effect of a cause of the exposure and a cause of the outcome), known as collider bias (Elwert & Winship, 2014; Hernan et al., 2004). Figures 1*E* and *F* show how bias can result from selective non-response or attrition in longitudinal studies. Figure 1*E* represents a longitudinal study examining the association between maternal smoking in pregnancy (*X*) and child autism (*Y*). Those with a mother who smoked in pregnancy (*X*) and males (*U*) are less likely to participate in the follow-up (*R*). If a male participant provides follow-up data, then it is less likely that the alternative cause of drop-out (maternal smoking in pregnancy) will be present. This results in a negative association between *X* (maternal smoking) and *U* (male gender) in those with complete outcome data. Male gender (*U*) is positively associated with child autism (*Y*), therefore, restricting to those with complete outcome data will result in the positive association between *X* (maternal smoking in pregnancy) and *Y* (child autism) being underestimated; see (Hernan et al., 2004) for an alternative example. Non-response or attrition results in bias when conditioning on response introduces a spurious path between the exposure and outcome (Elwert & Winship, 2014). Further examples of selection bias, including attrition, are described in detail elsewhere (Daniel, Kenward, Cousens, & De Stavola, 2012; Elwert & Winship, 2014; Hernan et al., 2004). *Measurement bias*. Measurement bias results from errors in assessment of the variables in the analysis due to imprecise data collection methods (for example, self-report measures of socially undesirable behaviours such as smoking can often be underreported). Measurement error can be either differential (e.g. measurement error in the exposure is related to the outcome or vice versa) or non-differential. With a few exceptions (e.g. non-differential measurement error in a continuous outcome) both non-differential and differential measurement error will result in bias (Hernan & Cole, 2009; Jiang & VanderWeele, 2015; VanderWeele, 2016). Figure 1*G* shows an example of non-differential measurement error in a mediator. *M* refers to the true mediator, M* refers to the measured mediator, and *U*_M_ refers to the measurement error for *M* (Hernan & Cole, 2009). Reducing measurement error is especially important in the context of a mediation model, because measurement error in the mediator often leads to an underestimated indirect effect and an overestimated direct effect (Blakely, McKenzie, & Carter, 2013; VanderWeele, 2016). Figure 1*H* shows an example of differential measurement error. Measurement error in the exposure *X* (parent smoking in pregnancy assessed retrospectively) is influenced by the outcome *Y* (child behavioural problems) resulting in bias in the exposure-outcome association. When there is measurement error in both the exposure and the outcome, it can be dependent (when the errors are associated, for example, due to measurement using a common instrument) or independent. Both differential measurement error and dependent measurement error can open a backdoor pathway between the exposure and outcome (Hernan & Cole, 2009).

Various statistical approaches exist that aim to minimize biases in observational data and can increase confidence to a certain degree. This section focuses on a few key approaches that are either frequently used or particularly relevant for research questions in mental health epidemiology. In Box 3 we discuss the importance of mechanisms, and the use of counterfactual mediation in the mental health literature.

Box 3. Mechanisms

**Table:** 

Mechanistic evidence can strengthen causal inference; indeed, some argue that causality cannot be established until a mechanism is identified (Glennan, 1996; Russo & Williamson, 2007). However, the causal role of certain exposures (for example, smoking in lung cancer) was largely accepted even before the underlying mechanisms were understood. Mediation analyses can be used to assess the relative magnitude of different pathways by which an exposure may affect an outcome. Traditional approaches to mediation, including the product-of-coefficients method (MacKinnon, Lockwood, Hoffman, West, & Sheets, 2002), are frequently used to examine mechanisms that may explain associations between an exposure and outcome in mental health research. More recently, *counterfactual mediation* (VanderWeele, 2015) is being increasingly used within the mental health literature (Aitken et al., 2018; Froyland, Bakken, & von Soest, 2020; Hammerton et al., 2020; Loret de Mola et al., 2020; Nguyen, Webb-Vargas, Koning, & Stuart, 2016). Although performing mediation analyses in a counterfactual framework is still subject to all the same threats to causal inference as traditional approaches to mediation analyses (including poorly measured or unmeasured confounding), it holds several advantages over traditional methods. First, the presence of an interaction between the exposure and mediator on the outcome can be tested. Second, binary mediators and outcomes can be included with effect estimates that are easily interpretable. Third, the counterfactual framework makes the assumptions regarding confounding much more explicit. Finally, it encourages the use of sensitivity analyses to examine the potential impact on conclusions of unmeasured confounding and measurement bias. VanderWeeele provides a methodological description (VanderWeele, 2015) and Krishna Rao and colleagues (Krishna Rao et al., 2015) provide an applied example using substance use. A further source of mechanistic evidence, which can provide support for causal claims within a triangulation framework, is so-called ‘incommensurable evidence’ – insights into plausible biological mechanisms that could explain a causal pathway between an exposure and an outcome. This can include evidence from model systems (e.g. rodent studies and human laboratory studies). In many cases, such evidence may be too far removed to allow direct comparison with evidence from epidemiological studies (and there are dangers associated with selecting evidence of this kind *post hoc*). However, in principle it may be powerful additional source of evidence, particularly if conceived prospectively.

In Table 1, we outline the assumptions and limitations for the main statistical approaches highlighted in this review and provide examples of each using mental health research.

**Table 1.** Assumptions and limitations of statistical and design-based approaches to causal inference

Statistical approaches	Description	Assumptions	Limitations	Example
Confounding				
Multivariable regression	Potential confounders are included in the regression model for the effect of the exposure on the outcome	No residual confounding (all confounders are accurately measured, and correctly included in the statistical model); for multivariable regression, the outcome is modelled correctly given the exposure and confounders, for propensity score methods the exposure is modelled correctly given the confounders	Assumptions difficult to meet with full confidence resulting in bias from residual confounding; although propensity scores carry some advantages over multivariable regression (e.g. statistical efficiency and flexibility), the different methods to incorporate a propensity score into the analysis model (e.g. stratifying, matching, adjusting, weighting) each have their own limitations – see Haukoos and Lewis (Haukoos & Lewis, 2015) for an overview	Harrison and colleagues (Harrison et al., 2020) performed a multivariable logistic regression between smoking behaviours and suicidal ideation and attempts, adjusting for potential confounders including age, sex and socio-economic position
Propensity scores	Propensity scores are used to control for time-invariant confounding, calculated by estimating the probability that an individual is exposed, given the values of their observed baseline confounders; can be extended to address time-varying confounding via marginal structural models			Bray and colleagues (Bray et al., 2019) used a propensity score to adjust for confounding when examining the association between reasons for alcohol use latent class membership during the year after high school and problem alcohol use at age of 35 years
Fixed-effects regression	This approach uses repeated measures of an exposure and an outcome to account for the possibility of an association between the exposure and the unexplained variability in the outcome (representing unmeasured confounding); can adjust for all time-invariant confounders, including unobserved confounders, and can incorporate observed time-varying confounders	Potential time-varying confounders are measured accurately and correctly included in the statistical model	Requires repeated assessments of exposure and outcome; model cannot control for unobserved fixed confounding factors whose effects vary with age, or that combine interactively with the exposure to influence the outcome, or unobserved time-varying confounders	Fergusson and Horwood (Fergusson & Horwood, 2000) used fixed-effects regression to assess the influence of deviant peer affiliations on substance use and crime across adolescence and young adulthood, taking into account unobserved fixed confounding factors and observed time-varying factors
Selection bias				
Complete case analysis with covariate adjustment	Analyses are performed on those with complete data on all variables, but covariates are included in the model that are associated with missingness	Data are MAR or MCAR; results can be unbiased when data are MNAR as long as the chance of being a complete case does not depend on the outcome after adjusting for covariates	Cannot address lack of power due to missing data; results biased when outcome MNAR; must be aware of and measure predictors of missingness; cannot include information from variables not included in main analysis that are associated with missingness	Hughes and colleagues (Hughes et al., 2019) use a hypothetical example examining the relationship between cannabis use at 15 years with depression symptoms and self-harm at age 21 years to describe missing mechanisms using causal diagrams and provide situations where complete case analysis and multiple imputation will or will not result in bias
Approaches based on the MAR assumption, e.g. multiple imputation	Multiple imputation is a two-stage process, where first, multiple imputed data sets are created with each missing value replaced by imputed values using models fitted to the observed data, and second, each imputed data set is analysed, and results are combined in an appropriate way; can address both lack of power and bias (with extensions that exist to allow for MNAR mechanisms using sensitivity parameters)	Data are MAR or MCAR; imputation model is compatible with analysis model; imputation is performed multiple times and performed ‘properly;’ final analysis combines appropriately over the multiple data sets (e.g. using Rubin's rules); for a more in-depth discussion of potential pitfalls in multiple imputation see the review by Sterne and colleagues (Sterne et al., 2009)	If exposure is MNAR, multiple imputation can cause more bias than using complete case analysis; requires information to be collected on auxiliary variables, closely associated with variables to be imputed; all aspects of the analysis model must be included in the imputation model, therefore if changes are made at a later date (e.g. testing an interaction), the imputation model needs to be redone; computationally intensive therefore can result in computational problems (particularly with small sample sizes)	
Approaches based on the MNAR assumption, e.g. using linkage to external routinely collected health records	Routinely collected health data can be used to examine biases from selective non-response by providing data on those that did and did not respond to assessments within population cohorts or surveys; it can also be used as a proxy for the missing study outcome in multiple imputation or deriving weights to adjust for potential bias and make the MAR assumption more plausible	High correlation between study outcome and linked proxy; if the outcome is not MNAR but missingness depends on the proxy, inclusion of the proxy in a multiple imputation model would increase bias – see Cornish and colleagues (Cornish et al., 2017) for an example)	Requires access to closely related routinely collected data; not all participants may consent to linkage which could introduce bias if differences between non-consenters and non-responders; linkage to external datasets can be costly and complicated; use of a proxy in multiple imputation can increase bias depending on missing data mechanism	Gorman and colleagues (Gorman et al., 2017) found that the use of routinely collected health data on alcohol-related harm in a multiple imputation model resulted in higher alcohol consumption estimates among Scottish men
Measurement bias				
Latent variables using multiple sources of data	A latent variable is a source of variance not directly measured but estimated from the covariation between a set of strongly related observed variables; if these observed variables are assessed using multiple methods, each with different sources of bias, variability due to bias shared across items can be removed from the latent variable	Latent variable indicators all measure same underlying construct and responses on the indicators are a result of an individual's position on the latent variable; latent variable variance is independent from measurement residual variance; indicators assessed using different methods have different sources of bias; for a description of all assumptions in latent variable modelling see Kline (Kline, 2015)	Requires at least four strongly correlated measures assessed using different methods each with different sources of bias; important that items included make theoretical sense given underlying construct; important to think carefully about the meaning of the latent variable	Palmer and colleagues (Palmer et al., 2002) describe a method using two self-report and two biochemical measures of smoking (carbon monoxide and cotinine), to remove variability due to self-report bias (e.g. recall or social desirability bias) and biological bias (e.g. second-hand smoke) and create a latent variable representing cigarette smoking
Mechanisms				
Counterfactual mediation	Mediation approach based on conceptualizing ‘potential outcomes’ for each individual [*Y*(*x*)] that would have been observed if particular conditions were met (i.e. had the exposure *X* been set to the value *x* through some intervention) – regardless of the conditions that were in fact met for each individual; allows the presence of an interaction between the exposure and mediator to be tested, inclusion of binary mediators and outcomes, and sensitivity analyses to examine potential impact on conclusions of unmeasured confounding and measurement bias	Main assumptions include conditional exchangeability, no interference and consistency; see de Stavola and colleagues (De Stavola, Daniel, Ploubidis, & Micali, 2015) for an accessible description of these assumptions and a comparison to assumptions made when estimating mediation within an SEM framework	Still subject to the same threats to causality as traditional approaches to mediation analyses (including poorly measured or unmeasured confounding and measurement error); challenging to extend to examine individual paths via multiple mediators; each specific counterfactual mediation method subject to its own limitations – see VanderWeele (VanderWeele, 2015)	Using a sequential counterfactual mediation approach, Aitken and colleagues (Aitken, Simpson, Gurrin, Bentley, & Kavanagh, 2018) showed that behavioural factors (including smoking and alcohol consumption) explained a further 5% of the association between disability acquisition and poor mental health in adults after accounting for material and psychosocial factors. The authors also performed a bias analysis which showed that the indirect effects were unlikely to be explained by unmeasured mediator-outcome confounding
Design-based approaches				
RCTs	In an RCT, participants are randomly assigned to a treatment or control group, and the outcome is compared across groups; when performed well, RCTs can account for both known and unknown confounders and are therefore considered to be the gold standard for estimating causal effects	Assignment to treatment and control groups is random, and so groups are similar except with respect to the intervention	Prone to potential bias, such as lack of concealment of the random allocation, failure to maintain randomization, lack of blinding to which group participants have been randomized, non-adherence, and differential loss to follow-up between groups; often recruit highly selected samples which are not representative of the population of interest, threatening the generalizability of results; can be expensive and time-consuming and not always feasible or ethical, particularly in mental health research	Ford and colleagues (Ford et al., 2019) performed a cluster RCT to examine the effectiveness and cost-effectiveness of the Incredible Years Teacher Classroom Management programme as a universal intervention in primary school children; the intervention reduced the total difficulties score on the Strength and Difficulties Questionnaire at 9 months compared to teaching as usual, but this did not persist at 18 or 30 months
Natural experiments	Populations are compared before and after (or with and without exposure to) a ‘natural’ exposure at a specific time point, with the assumption that potential biases (such as confounding) are similar between them; exposure may occur naturally (e.g. famine), or be quasi-random (e.g. introduction of policies)	Populations compared are comparable (e.g. with respect to the underlying confounding structure) except for the naturally occurring (or quasi-randomized) exposure	Potential sources of bias include differences on characteristics that may confound any observed association, or misclassification of outcome that relates to the naturally occurring exposure; relies on the occurrence of appropriate natural experiments that manipulate exposure of interest; selection bias can be present as exposure is not manipulated by researcher	Davies and colleagues (Davies et al., 2018a) used the raising of the school leaving age from 15 to 16 years as a natural experiment for testing whether remaining in school at 15 years of age affected later health outcomes (including depression diagnosis, alcohol use and smoking)
Instrumental variables	An instrumental variable is a variable that is robustly associated with an exposure of interest, but not confounders of the exposure and outcome. MR is an extension of this approach where a genetic variant is used as a proxy for the exposure	The instrument is associated with the exposure (relevance assumption); the instrument is not associated with confounders of the exposure-outcome association (exchangeability assumption); the instrument is not associated with the outcome other than via its association with the exposure (exclusion restriction assumption)	Weak instrument bias can result from a weak association between the instrument and the exposure; another source of bias is the exclusion restriction criterion being violated – this is the main source of bias in MR (due to horizontal pleiotropy), and therefore a number of extensions have been developed which are robust to horizontal pleiotropy; population stratification is also a source of bias in MR, which may require focusing on an ethnically homogeneous population, or adjusting for genetic principal components that reflect different population sub-groups	Taylor and colleagues (Taylor et al., 2020) used the tendency of physicians to prefer prescribing one medication over another as an instrumental variable in testing the association between varenicline (*v*. nicotine replacement therapy) with smoking cessation and mental health
Different confounding structures	Multiple samples with different confounding structures are used, for example, comparing multiple control groups within a case−control design, or multiple populations with different confounding structures	The bias introduced by confounding is different across samples so that congruent results are more likely to reflect causal effects; different results across samples are due to different confounding structures and not true differences in causal effect; no other sources of bias that could explain results being the same or different across samples	Assessment and quality of measures must be similar across samples; misclassification of exposure or outcome (or other unknown sources of bias) can produce misleading results; strong a priori hypotheses required about confounding structures across samples	Sellers and colleagues (Sellers et al., 2020) compared the association between maternal smoking in pregnancy and offspring birth weight, cognition and hyperactivity in two national UK cohorts born in 1958 and 2000/2001 with different confounding structures
Positive and negative controls	This approach allows a test of whether an exposure or outcome is behaving as expected (a positive control), or not as expected (a negative control); a positive control is known to be causally related to the outcome (or exposure), whereas a negative control is not plausibly causally related to outcome (or exposure)	The real exposure (or outcome) and negative control exposure (or outcome) have the same sources of bias; the negative control exposure is not causally related to the outcome (and vice versa for negative control outcome); the positive control exposure is causally related to the outcome (and vice versa for positive control outcome)	Important to consider assortative mating in the prenatal negative control design, and mutually adjust for maternal and paternal exposures [see Madley-Dowd and colleagues (Madley-Dowd et al., 2020b)]; appropriate negative control variables can be difficult to identify (e.g. where an exposure may have diverse effects on a range of outcomes)	Caramaschi and colleagues (Caramaschi et al., 2018) used paternal smoking during pregnancy as a negative control exposure to investigate whether the association between maternal smoking during pregnancy and offspring autism is likely to be causal, on the assumption that any biological effect of paternal smoking on offspring autism will be negligible, but that confounding structures will be similar to maternal smoking
Discordant siblings	Family-based study designs can provide a degree of control over family-level confounding by comparing outcomes for siblings who are discordant for an exposure; for example, two siblings born to a mother who smoked during one pregnancy, but not the other, provide information on the intrauterine effects of tobacco exposure, while controlling for observed and unobserved genetic and shared environmental familial confounding	Any misclassification of the exposure or outcome is similar across siblings, and there is little or no individual-level confounding (for example, one sibling was not exposed to a potential confounder where the other was not)	The assumption of no individual-level confounding is unlikely to be met (for example, the plausible scenario where a mother is both older and less likely to be smoking for the second pregnancy); method depends on the availability of suitable samples which means sample size can be limited (particularly for use of identical twins within a discordant-sibling design); bias due to individual-level confounding or misclassification of exposure/ outcome will be larger than in studies of unrelated individuals – see Frisell and colleagues (Frisell, Oberg, Kuja-Halkola, & Sjolander, 2012)	Madley-Dowd and colleagues (Madley-Dowd et al., 2020a) used a Danish cohort of parents and siblings to examine the association between maternal smoking in pregnancy and offspring intellectual disability; the lack of within-family effect suggested that any association was due to genetic or environmental confounders shared between the siblings; a positive control outcome (birthweight) where a causal relation with the exposure (maternal smoking in pregnancy) is well established was used to validate the method

MAR, missing at random; MCAR, missing completely at random; MNAR, missing not at random; SEM, structural equation modelling; RCT, randomized controlled trial; MR, Mendelian randomization.

### Confounding and reverse causality

The most common approach to address confounding bias is to include any confounders in a regression model for the effect of the exposure on the outcome. Alternative methods to address either time-invariant confounding (e.g. *propensity scores*) or time-varying confounding (e.g. *marginal structural models*) are increasingly being used in the field of mental health (Bray, Dziak, Patrick, & Lanza, 2019; Howe, Cole, Mehta, & Kirk, 2012; Itani et al., 2019; Li, Evans, & Hser, 2010; Slade et al., 2008; Taylor et al., 2020). However, these approaches all rely on all potential confounders being measured and no confounders being measured with error. These are typically unrealistic assumptions when using observational data, resulting in the likelihood of residual confounding (Phillips & Smith, 1992). Ohlsson and Kendler provide a more in-depth review of the use of these methods in psychiatric epidemiology (Ohlsson & Kendler, 2020).

Another approach to address confounding is fixed-effects regression; for a more recent extension to this method, see (Curran, Howard, Bainter, Lane, & McGinley, 2014). Fixed-effects regression models use repeated measures of an exposure and an outcome to account for the possibility of an association between the exposure and the unexplained variability in the outcome (representing unmeasured confounding) (Judge, Griffiths, Hill, & Lee, 1980). These models adjusted for all time-invariant confounders, including unobserved confounders, and can incorporate observed time-varying confounders. This method has been described in detail elsewhere – see (Fergusson & Horwood, 2000; Fergusson, Swain-Campbell, & Horwood, 2002) – and fixed-effects regression models have been used to address various mental health questions, including the relationship between alcohol use and crime (Fergusson & Horwood, 2000), cigarette smoking and depression (Boden, Fergusson, & Horwood, 2010), and cultural engagement and depression (Fancourt & Steptoe, 2019).

### Selection bias

One of the most common types of selection bias present in observational data is from selective non-response and attrition. Conventional approaches to address this potential bias (and loss of power) include multiple imputation, full information maximum likelihood estimation, inverse probability weighting, and covariate adjustment. Comprehensive descriptions of these methods are available (Enders, 2011; Seaman & White, 2013; Sterne et al., 2009; White, Royston, & Wood, 2011). In general, these approaches assume that data are *missing at random* (MAR); however, missing data relating to mental health are likely to be *missing not at random* (MNAR). In other words, the probability of *Z* being missing still depends on unobserved values of *Z* even after allowing for dependence on observed values of *Z* and other observed variables. Introductory texts on *missing data mechanisms* are available (Graham, 2009; Schafer & Graham, 2002). An exception to this is using complete case analysis, with covariate adjustment which can be unbiased when data are MNAR as long as the chance of being a complete case does not depend on the outcome after adjusting for covariates (Hughes, Heron, Sterne, & Tilling, 2019). Additionally, extensions to standard multiple imputation exist that allow for MNAR mechanisms using sensitivity parameters (Leacy, Floyd, Yates, & White, 2017; Tompsett, Leacy, Moreno-Betancur, Heron, & White, 2018).

Further approaches to address potential MNAR mechanisms include linkage to external data (Cornish, Macleod, Carpenter, & Tilling, 2017; Cornish, Tilling, Boyd, Macleod, & Van Staa, 2015), MNAR analysis models for longitudinal data (Enders, 2011; Muthen, Asparouhov, Hunter, & Leuchter, 2011) and sensitivity analyses (Leacy et al., 2017; Moreno-Betancur & Chavance, 2016). Linkage to routinely collected health data is starting to be used in the context of mental health (Christensen, Ekholm, Gray, Glumer, & Juel, 2015; Cornish et al., 2015; Gorman et al., 2014; Gray et al., 2013; Mars et al., 2016) to examine the extent of biases from selective non-response by providing data on those that did and did not respond to assessments within population cohorts or health surveys. In addition to using linked data to detect potential non-response bias, it can also be used as a proxy for the missing study outcome in multiple imputation or deriving weights to adjust for potential bias and make the assumption of MAR more plausible (Cornish et al., 2015, 2017; Gorman et al., 2017; Gray et al., 2013).

### Measurement bias

Conventional approaches to address measurement error include using *latent variables*. Here, when we use the term measurement error, we are specifically referring to variability in a measure that is not due to the construct that we are interested in. Using a latent variable holds several advantages over using an observed measure that represents a sum of the relevant items, for example, allowing each item to contribute differently to the underlying construct (via factor loadings) and reducing measurement error (Muthen & Asparouhov, 2015). However, if the source of measurement error is shared across all the indicators (for example, when using multiple self-report questions), the measurement error may not be removed from the construct of interest. Various extensions to latent variable methods have been developed to specifically address measurement bias from using self-report questionnaires. For example, using items assessed with multiple methods, each with different sources of bias (such as self-report and objective measures), means that variability due to bias shared across particular items can be removed from the latent variable representing the construct of interest. For an example using cigarette smoking see Palmer and colleagues (Palmer, Graham, Taylor, & Tatterson, 2002). Alternative approaches to address measurement error in a covariate exist, but will not be discussed further here, including regression calibration (Hardin, Schmiediche, & Carroll, 2003; Rosner, Spiegelman, & Willett, 1990) and the simulation extrapolation method (Cook & Stefanski, 1994; Hardin et al., 2003; Stefanski & Cook, 1995).

### Conclusions

Various advanced statistical approaches exist that bring certain advantages in terms of addressing biases present in observational data. These approaches are easily accessible and are starting to be used in the field of mental health. Most commonly, these approaches are applied in isolation, or sequentially to account for a combination of bias due to confounding, selection and measurement. However, other methods also exist that use models to simultaneously address all three types of bias – van Smeden and colleagues (van Smeden, Penning de Vries, Nab, & Groenwold, 2020) provide a review on these types of biases. The first step in causal inference with observational data is to identify and measure the important confounders and include them correctly in the statistical model. This process can be facilitated using causal diagrams (Box 2). However, even when studies have measured potential confounders extensively, there could still be some bias from residual confounding because of measurement error. In practice, these statistical approaches cannot always completely remove key sources of bias; therefore, using design-based approaches to improve causal inference (outlined below) is also important.

## Design-based approaches to causal inference

A fundamentally different approach to causal inference is to use design-based approaches, rather than statistical approaches that attempt to minimize or remove sources of bias (e.g. by adjustment for potential confounders). Here it is the design of the study that addresses the problem of potential bias – either by ensuring it is not present (under certain assumptions), or by comparing results across methods with different sources and direction of potential bias (Richmond, Al-Amin, Davey Smith, & Relton, 2014). This final point will be returned to when we discuss *triangulation* of results. In Table 1, we outline the assumptions and limitations of each design-based approach, and provide specific examples drawn from the mental health literature. For further examples of the use of natural experiments in psychiatric epidemiology see the review by Ohlsson and Kendler (Ohlsson & Kendler, 2020).

### Randomized controlled trials

The RCT is typically regarded as the most robust basis for causal inference and represents the most common approach that uses study design to support the causal inference. Nevertheless, RCTs rest on the critical assumption that the groups are similar except with respect to the intervention. If this assumption is met, the exposed and unexposed groups are considered exchangeable, which is equivalent to observing the outcome that would occur if a person were exposed, and what would occur if they were not exposed. An RCT is also still prone to potential bias, such as lack of concealment of the random allocation, failure to maintain randomization, and differential loss to follow-up between groups. These sources of bias are typically addressed through the application of robust randomization and other study procedures. Further limitations include that RCTs are not always feasible, and often recruit highly selected samples (e.g. for safety considerations, or to ensure high levels of compliance), so the generalizability of results from RCTs can be an important limitation.

### Natural experiments

Where RCTs are not practical or ethical, natural experiments can provide an alternative. These compare populations before and after a ‘natural’ exposure, leading to ‘quasi-random’ exposure (e.g. using *regression discontinuity* analysis). The key assumption is that the populations compared are comparable (e.g. with respect to the underlying confounding structure) except for the naturally occurring exposure. Potential sources of bias include differences in characteristics that may confound any observed association or misclassification of the exposure that relates to the naturally occurring exposure. This approach also relies on the occurrence of appropriate natural experiments that manipulate the exposure of interest (e.g. policy changes that mandate longer compulsory schooling, resulting in an increase in years of education from one cohort to another) (Davies, Dickson, Davey Smith, van den Berg, & Windmeijer, 2018a).

### Instrumental variables

In the absence of an appropriate natural experiment, an alternative is to identify an instrumental variable that can be used as a proxy for the exposure of interest. An instrumental variable is a variable that is robustly associated with an exposure of interest but is not a confounder of the exposure and outcome. For example, the tendency of physicians to prefer prescribing one medication over another (e.g. nicotine replacement therapy v. varenicline for smoking cessation) has been used as an instrument in pharmacoepidemiological studies (Itani et al., 2019; Taylor et al., 2020). The key assumption is that the instrument is not associated with the outcome other than that via its association with the exposure (the exclusion restriction assumption). Other assumptions include the relevance assumption (that the instrument has a causal effect on the exposure), and the exchangeability assumption (that the instrument is not associated with potential confounders of the exposure–outcome relationship). Potential sources of bias include the instrument not truly being associated with the exposure, or the *exclusion restriction criterion* being violated. If the association of the instrument with the exposure is weak this may lead to so-called weak instrument bias (Davies, Holmes, & Davey Smith, 2018b), which may, for example, amplify biases due to violations of other assumptions (Labrecque & Swanson, 2018). This can be a particular problem in genetically informed approaches such as Mendelian randomization (MR) (see below), where genetic variants typically only predict a small proportion of variance in the exposure of interest. A key challenge with this approach is testing the assumption that the instrument is not associated with the outcome via other pathways, which may not always be possible. More detailed descriptions of the instrumental variable approach, including the underlying assumptions and potential pitfalls, are available elsewhere (Labrecque & Swanson, 2018; Lousdal, 2018).

### Different confounding structures

If it is not possible to use design-based approaches that (in principle) are protected from confounding, an alternative is to use multiple samples with different confounding structures. For example, multiple control groups within a case−control design, where bias for the control groups is in different directions, can be used under the assumption that if the sources of bias in the different groups are indeed different, this would produce different associations, whereas a causal effect would produce the same observed association. A related approach is the use of cross-context comparisons, where results across multiple populations with different confounding structures are compared, again on the assumption that the bias introduced by confounding will be different across contexts so that congruent results are more likely to reflect causal effects. For example, Sellers and colleagues (Sellers et al., 2020) compared the association between maternal smoking in pregnancy and offspring birthweight, cognition and hyperactivity in two national UK cohorts born in 1958 and 2000/2001 with different confounding structures.

### Positive and negative controls

The use of positive and negative controls – common in fields such as preclinical experimental research – can be applied to both exposures and outcomes in observational epidemiology. This allows us to test whether an exposure or outcome is behaving as we would expect (a positive control), and as we would *not* expect (a negative control). A positive control exposure is one that is known to be causally related to the outcome and can be used to ensure the population sampled generates credible associations that would be expected (i.e. is not unduly biased), and vice versa for a positive control outcome. A negative control exposure is one that is *not* plausibly causally related to the outcome, and again vice versa for a negative control outcome. For example, smoking is associated with suicide, which is plausibly causal but is also equally strongly associated with homicide, which is not. The latter casts doubt on a causal interpretation of the former (Davey Smith, Phillips, & Neaton, 1992). Brand and colleagues (Brand et al., 2019) used paternal smoking during pregnancy as a negative control exposure to investigate whether the association between maternal smoking during pregnancy and foetal growth is likely be causal, on the assumption that any biological effects of paternal smoking on foetal growth will be negligible, but that confounding structures will be similar to maternal smoking. Overall, negative controls provide a powerful means by which the assumptions underlying a particular approach (e.g. that confounding has been adequately dealt with) can be tested, although in some cases identifying an appropriate negative control can be challenging (e.g. where exposure may have diverse effects on a range of outcomes). Lipsitch and colleagues (Lipsitch, Tchetgen Tchetgen, & Cohen, 2010) described their use as a means whereby we can ‘detect both suspected and unsuspected sources of spurious causal inference’. In particular, negative controls can be used in conjunction with most of the methodologies we discuss here – for example, negative controls can be used to test some of the assumptions of an instrumental variable or genetically informed approaches. For example, there is evidence that genetic variants associated with smoking may also be associated with outcomes at age 7, prior to exposure to smoking, which provides reasons to be cautious when using these variants as proxies for smoking initiation in MR (see below) (Khouja, Wootton, Taylor, Davey Smith, & Munafo, 2020). Madley-Dowd and colleagues (Madley-Dowd, Rai, Zammit, & Heron, 2020b) provide an accessible introduction to the prenatal negative control design and the importance of considering assortative mating, explained using *causal diagrams*, whereas Lipsitch and colleagues (Lipsitch et al., 2010) provide a more general review of the use of negative controls in epidemiology.

### Discordant siblings

Family-based study designs can provide a degree of control over family-level confounding. For example, two siblings born to a mother who smoked during one pregnancy, but not the other, provide information on the intrauterine effects of tobacco exposure while controlling for observed and unobserved familial confounding (both genetic and environmental), including shared confounders and 50% of genetic confounding. This approach assumes that any misclassification of the exposure or the outcome is similar across siblings, and there is little or no individual-level confounding, an assumption that is often not met (e.g. in the plausible scenario where a mother is both older and less likely to be smoking for the second pregnancy). An extension of this approach is the use of identical twins within a discordant-sibling design, which controls for 100% of genetic confounding (Keyes, Davey Smith, & Susser, 2013). An advantage of this approach is that does not require the direct measurement of genotype, but it depends on the availability of suitable samples. This can mean that the sample size may be limited. Pingault and colleagues (Pingault et al., 2018) describe a range of genetically informed approaches in more detail, including family-based designs such as the use of sibling and twin designs.

### Genetically informed approaches

MR is a now a widely used genetically informed design-based method for causal inference, which is often implemented through an instrumental variable analysis (Richmond & Davey Smith, 2020). MR is generally implemented through the use of genetic variants as proxies for the exposure of interest (Davey Smith & Ebrahim, 2003; Davies et al., 2018b). For example, Harrison and colleagues (Harrison, Munafo, Davey Smith, & Wootton, 2020) used genetic variants associated with a range of smoking behaviours as proxies to examine the effects of smoking on suicidal ideation and suicide attempts. Violation of the exclusion restriction criterion due to horizontal (or biological) *pleiotropy* is the main likely source of bias, and for this reason, a number of extensions to the foundational method have been developed that are robust to horizontal pleiotropy (Hekselman & Yeger-Lotem, 2020; Hemani, Bowden, & Davey Smith, 2018). *Population stratification* is another potential source of bias, which may require focusing on an ethnically homogeneous population, or adjusting for genetic principal components that reflect different population sub-groups. Weak instrument bias (see above) is also a common problem in MR (although often underappreciated), given that genetic variants often only account for a small proportion of variance in the exposure of interest. Diemer and colleagues (Diemer, Labrecque, Neumann, Tiemeier, & Swanson, 2020) describe the reporting of methodological limitations of MR studies in the context of prenatal exposure research and find that weak instrument bias is reported less often as a potential limitation than pleiotropy or population stratification. MR approaches can be extended to include comparisons across context, the use of positive and negative controls, and the use of family-based designs (including discordant siblings). More detailed reviews of a range of genetically informed approaches, including MR, are available elsewhere (Davies et al., 2019; Pingault et al., 2018).

### Conclusions

A variety of design-based approaches to causal inference exist that should be considered complementary to statistical approaches. In particular, several of these approaches (e.g. analyses across groups with different confounding structures, and the use of positive and negative controls) can be implemented using the range of statistical methods described above. These are again increasingly being used in the field of mental health. However, despite their strengths, it is unlikely that any single method (whether statistical or design-based) can provide a definite answer to a causal question.

## Triangulation and causal inference

One reason to include design-based approaches is that these may be less likely to suffer from similar sources and directions of bias compared with statistical approaches, particularly when these are conducted within the same data set (Lawlor, Tilling, & Davey Smith, 2016). Ideally, we would identify different sources of evidence that we could apply to a research question and understand the likely sources and directions of bias operating within each so that we could ensure that these are different. This means that *triangulation* should be a prospective approach, rather than simply selecting sources of evidence that support a particular conclusion post hoc.

A range of examples of studies that explicitly use triangulation to support stronger causal inference in the context of substance use and mental health is presented in Table 2. Although this is not an exhaustive list of studies that have used triangulation in mental health research, we identified several studies by searching (i) for studies that cited a review on triangulation in aetiological epidemiology from 2017 (Lawlor et al., 2016), (ii) two databases (PubMed and Web of Science) in March 2020 using the search terms ‘triangulat*’ and ‘mental health’ for papers published since 2017 and (iii) the reference list of another recent review on triangulation of evidence in genetically informed designs (Munafo, Higgins, & Davey Smith, 2020). For a description of two additional studies in psychiatric epidemiology that have used a triangulation framework see the review by Ohlsson and Kendler (Ohlsson & Kendler, 2020). These studies use a range of statistical and design-based approaches. For example, Caramaschi and colleagues (Caramaschi et al., 2018) explore the impact of maternal smoking during pregnancy on offspring autism spectrum disorder (ASD), using paternal smoking during pregnancy as a negative control, and MR using genetic variants associated with heaviness of smoking as a proxy for the exposure, together with conventional regression-based analyses. The evidence was not consistent with a causal effect for maternal smoking in pregnancy on ASD.

**Table 2.** Studies using triangulation to address a research question in mental health epidemiology

Study	Exposure	Outcome	Approach used	Description	Comments
Brand et al. (2019)	Maternal smoking in pregnancy	Longitudinal foetal growth from 12–16 to 40 weeks gestation	Linear regression	Multilevel fractional polynomial models of estimated foetal weight, and multivariable linear regression between maternal smoking in pregnancy and foetal weight, adjusting for potential confounders	The study states that findings were triangulated from three approaches with differing sources of bias to improve causal inference; evidence was consistent with a causal effect for maternal smoking in pregnancy on foetal growth (i.e. results from all three methods were consistent with a causal effect)
			MR	MR of smoking quantity and ease of quitting on estimated foetal weight using individual-level data	
			Negative control exposure	Partner's smoking was used as a negative control for intrauterine exposure	
Thapar et al. (2009)	Maternal smoking in pregnancy	Child Attention Deficit/Hyperactivity Disorder (ADHD) and birth weight	Natural experiment	Natural experiment comparing offspring conceived via *in vitro* fertilization, who were either genetically related (fertilized eggs implanted in the biological mother) or genetically unrelated (fertilized eggs implanted in a surrogate mother) to the woman who underwent the pregnancy	Study does not specifically refer to triangulation; evidence was consistent with a causal effect for maternal smoking in pregnancy on lower birth weight but not ADHD symptoms (i.e. consistent results were found for unrelated and related mother–offspring pairs for birth weight but not ADHD)
Sellers et al. (2020)	Maternal smoking in pregnancy	Child conduct and hyperactivity, cognition and birth weight	Cross-cohort design	Two national UK cohorts born in 1958 and 2000/2001 with different confounding structures were compared	The study highlights the utility of cross-cohort designs in helping triangulate conclusions about the role of putative causal risk factors in observational epidemiology; evidence was consistent with a causal effect for maternal smoking in pregnancy on lower birth weight but not the other child outcomes (i.e. consistent results were found across cohorts for birth weight but not conduct problems, hyperactivity and reading)
Caramaschi et al. (2018)	Maternal smoking in pregnancy	Autism spectrum disorder (ASD)	Logistic and linear regression	Multivariable regression using self-report smoking and an epigenetic score as the exposure and ASD diagnosis or traits as the outcome, adjusted for potential confounders	Study states that the integration of evidence from several different epidemiological approaches that have differing and unrelated sources of bias was used, but does not specifically refer to triangulation; evidence was not consistent with a causal effect for maternal smoking in pregnancy on autism or related traits (i.e. all three methods showed weak or no evidence for a causal effect)
			Negative control exposure	Partner's smoking was used as a negative control for intrauterine exposure	
			MR	MR between heaviness of smoking and ASD or autistic traits using individual-level data	
Gage et al. (2020)	Smoking	Education attainment and cognitive ability	Linear regression	Multivariable linear regression between smoking heaviness and education attainment and cognitive ability, adjusting for potential confounders and earlier measures of the outcome	Study highlights that the triangulation of results across different methods, each with their own strengths, limitations and sources of bias is a strength; evidence was consistent with a causal effect for smoking on lower educational attainment, but results were less consistent for cognitive ability (i.e. results from both methods were consistent with a causal effect for education and cognition, however cognition results were less robust to various sensitivity analyses)
			MR	Two-sample MR of two smoking phenotypes (smoking initiation and lifetime smoking) on cognitive ability and educational attainment	
Harrison et al. (2020)	Smoking behaviours (initiation, smoking status, heaviness, lifetime smoking)	Suicidal ideation and attempts	Logistic regression	Multivariable logistic regression between smoking behaviours and suicidal ideation and attempts, adjusting for potential confounders	Study states that they triangulated across multiple methods, multiple smoking behaviours and multiple suicidal behaviours to improve causal inference; evidence was not consistent with a causal effect for smoking on suicidal ideation and attempts (i.e. an association was found in observational analyses but not MR)
			MR	Two-sample MR of smoking initiation on suicide attempt using five different MR methods; MR of lifetime smoking behaviour on suicidal ideation and attempt using individual-level data	
Itani et al. (2019)	Prescription of varenicline *v*. Nicotine replacement therapy (NRT)	Smoking cessation at 2-years	Logistic regression	Multivariable logistic regression between varenicline prescription and smoking cessation, adjusting for potential confounders both in those with and those without a neuro-developmental disorder	Study highlights that triangulating three different analytical methods to address confounding is a strength; evidence was consistent with a causal effect for varenicline on smoking cessation (i.e. results from all three methods were consistent with a causal effect)
			Propensity score matching	Participants were matched based on the association between their exposure and all baseline characteristics	
			Instrumental variable analysis	Physicians’ previously recorded prescribing preferences for varenicline *v*. NRT was used as the instrument	
Taylor et al. (2020)	Prescription of varenicline *v*. NRT	Smoking cessation and mental health	Logistic regression	Multivariable logistic regression between varenicline prescription and smoking cessation and mental health outcomes adjusting for potential confounders both in those with and those without a mental disorder	Study states that results were triangulated from three analytical techniques; evidence was consistent with a causal effect for varenicline on smoking cessation (i.e. results from all three methods were consistent with a causal effect); this study is not independent from Itani et al. (2019) above
			Propensity score matching	Participants were matched based on the association between their exposure and all baseline characteristics	
			Instrumental variable analysis	Physicians’ previously recorded prescribing preferences for varenicline *v*. NRT was used as the instrument	
Davies et al. (2018a)	Remaining in school	Various health outcomes including depression diagnosis, alcohol use and smoking	Natural experiment	The raising of the school leaving age from 15 to 16 years was used as a natural experiment for testing whether remaining in school at 15 years of age affected later outcomes; data analysed using a regression discontinuity design, instrumental variable analysis and difference-in-difference analysis	Study does not refer to triangulation; evidence was consistent with a causal effect for remaining in school on reduced diabetes and mortality (i.e. results from all three methods were consistent with a causal effect)
Sanderson, Davey Smith, Bowden, & Munafo (2019)	Educational attainment	Smoking behaviour (current smoking, smoking initiation and smoking cessation)	Logistic regression	Multivariable logistic regression between educational attainment and smoking behaviours, adjusting for general cognitive ability and potential confounders	Study states that results were compared within a triangulation framework; evidence was consistent with a causal effect for more years of education on smoking behaviour (i.e. results from both methods were consistent with a causal effect)
			MR	Multivariable MR of educational attainment and general cognitive ability on smoking behaviour using individual-level data; univariable and multivariable two-sample MR of educational attainment and general cognitive ability on smoking initiation and cessation	
Fancourt & Steptoe (2019)	Cultural engagement	Depression	Logistic regression	Multivariable regression between cultural engagement and depression, adjusting for potential confounders related to socio-economic status (SES) and baseline depression symptoms	Study states that a statistical triangulation approach was used, running three separate sets of analyses that each have different strengths and address different statistical limitations or biases; evidence was consistent with a causal effect for cultural engagement on depression (i.e. results from all three methods were consistent with a causal effect)
			Propensity score matching	Participants were matched based on the association between their exposure and SES	
			Fixed-effects regression	Regression model which takes account of all time-invariant factors (which include multiple aspects of SES) even if unobserved	

The limitations of observational data for causal inference are well known. However, the thoughtful application of multiple statistical and design-based approaches, each with their own strengths and weaknesses, and in particular sources and directions of bias, can support stronger causal inference through the triangulation of evidence provided by these. Triangulation can be within broad methods (e.g. propensity score matching and fixed-effects regression within regression-based statistical approaches, or different pleiotropy-robust MR methods), but is most powerful when it draws on fundamentally different methods, as this is most likely to ensure that sources of bias are different, and operating in different directions. It will be strongest when applied prospectively. This could in principle include the pre-registration of a triangulation strategy. This will encourage new research that does not simply have the same strengths and limitations as prior studies, but instead intentionally has a different configuration of strengths and limitations, and different sources (and, ideally, direction) of potential bias. It is also worth noting that triangulation is currently largely a qualitative exercise, although methods are being developed to support the quantitative synthesis of estimates provided by different methods.

Although triangulation is beginning to be applied in the context of mental health, our review of recent studies that explicitly make reference to triangulation revealed relatively few that did so. Of course, others will have included multiple approaches without describing the approach as one of triangulation, but it is in part this explicit (and ideally prospective) recognition of the need to understand potential sources of bias associated with these different methods that is a key. Our hope is that this approach will become more widely adopted – resulting in weightier outputs that provide more robust answers to key questions. This will have other implications – for example, larger teams of researchers contributing distinct elements to studies will become more common, and these contributions will need to be recognized in ways that conventional authorship does not fully capture. Triangulation can therefore be considered part of wider efforts to improve the transparency and robustness of scientific research, and the wider scientific infrastructure and system of incentives. Ultimately, we must always be cautious when attempting to infer causality from observational data. However, there are clear examples where causality was confirmed, even before the underlying mechanisms were well understood (e.g. smoking and lung cancer). In many respects, these conclusions might be considered the result of the accumulation of evidence from multiple sources – a triangulation of a kind. However, in our view, the adoption of a prospective and explicit triangulation framework offers the potential to accelerate progress to the point where we feel more confident in our causal inferences.
