# Causal inference with observational data: the need for triangulation of evidence – CORRIGENDUM

**DOI:** 10.1017/S0033291721002634

**Published:** 2021-07

**Authors:** Gemma Hammerton, Marcus R. Munafò

This article was published in Psychological Medicine with an error in Table 1, the correct version of Table 1 can be seen below.

**Figure:**
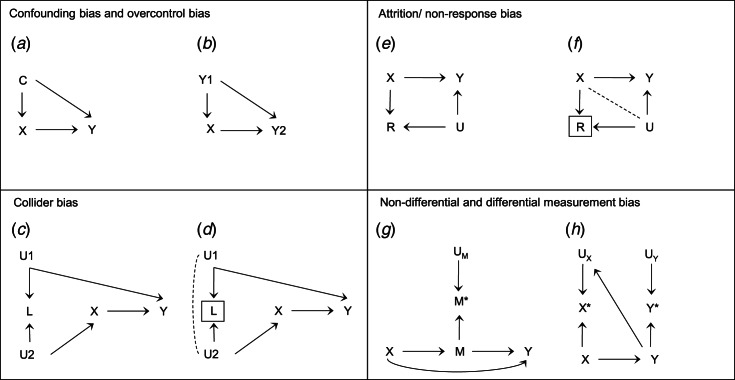

The authors apologise for this error.
